# Supplementary material for: Artificial intelligence-based methods for fusion of electronic health records and imaging data
Source: Sci Rep. 2022 Oct 26;12:17981. doi: 10.1038/s41598-022-22514-4 (PMC9605975; doi:10.1038/s41598-022-22514-4)
Supplement: Supplementary file 1 — Supplementary Information 1. [file 41598_2022_22514_MOESM1_ESM.docx]

**Appendix 1: List of Query used in various databases**

| **Database** | | **Query** | | |
| --- | --- | --- | --- | --- |
| **PubMed** | | ("machine learning"[Title/Abstract] OR "deep learning"[Title/Abstract] OR "artificial intelligence"[Title/Abstract] OR "neural network*" [Title/Abstract] OR "Convolutional neural network*" [Title/Abstract] OR "reinforcement learning" [Title/Abstract] OR "transfer learning" [Title/Abstract] OR "Artificial neural network*" [Title/Abstract] OR "supervised learning"[Title/Abstract] OR "unsupervised learning"[Title/Abstract] OR "ensemble learning" [Title/Abstract] OR "Support vector machine" [Title/Abstract] OR "Naïve Bayes" [Title/Abstract] OR "decision tree" [Title/Abstract] OR "Random Forest" [Title/Abstract] OR "Naive Bayes" [Title/Abstract] OR "Fuzzy Logic" [Title/Abstract] OR "K- Nearest Neighbor*" [Title/Abstract] OR "K-means" [Title/Abstract] OR "Long Short-Term Memory Networks" [Title/Abstract]) AND ("multimodal data" [Title/Abstract] OR "late fusion" [Title/Abstract] OR "joint fusion" [Title/Abstract] OR "early fusion" [Title/Abstract] OR "multimodality fusion" [Title/Abstract] OR "multimodal imaging" [Title/Abstract] OR "hybrid data" [Title/Abstract] OR "multimodal feature*" [Title/Abstract] OR "combined data" [Title/Abstract]) AND ("medical imag*"[Title/Abstract] OR "electronic health records"[Title/Abstract] OR "clinical data"[Title/Abstract] OR "MRI"[Title/Abstract] OR "fMRI"[Title/Abstract] OR "MR image*"[Title/Abstract] OR "sMRI"[Title/Abstract] OR "magnetic resonance imaging" [Title/Abstract] OR "EHR"[Title/Abstract] OR "CT"[Title/Abstract] OR "X-ray"[Title/Abstract] OR "ultrasound"[Title/Abstract] OR "Mammogram"[Title/Abstract]) AND (2015:2022[pdat])) AND (english[Filter])  -230 Results | | |
| **Scopus** | | ( TITLE-ABS-KEY ( "machine learning" ) OR TITLE-ABS-KEY ( "deep learning" ) OR TITLE-ABS-KEY ( "artificial intelligence" ) OR TITLE-ABS-KEY ("neural network*") OR TITLE-ABS-KEY ("Convolutional neural network*") OR TITLE-ABS-KEY ("reinforcement learning") OR TITLE-ABS-KEY ("transfer learning") OR TITLE-ABS-KEY ("supervised learning") OR TITLE-ABS-KEY ("unsupervised learning") OR TITLE-ABS-KEY ("ensemble learning") OR TITLE-ABS-KEY ("Support vector machine") OR TITLE-ABS-KEY ("Artificial neural network*") OR TITLE-ABS-KEY ("decision tree") OR TITLE-ABS-KEY ("Random Forest") OR TITLE-ABS-KEY ("Naïve Bayes") OR TITLE-ABS-KEY ("Naive Bayes") OR TITLE-ABS-KEY ("Fuzzy Logic") OR TITLE-ABS-KEY ("K- Nearest Neighbor*") OR TITLE-ABS-KEY ("K-means") OR TITLE-ABS-KEY ("Long Short-Term Memory Networks")) AND (TITLE-ABS-KEY ("multimodal data") OR TITLE-ABS-KEY ("late fusion") OR TITLE-ABS-KEY ("joint fusion") OR TITLE-ABS-KEY ("early fusion") OR TITLE-ABS-KEY ("multimodality fusion") OR TITLE-ABS-KEY ("multimodal imaging") OR TITLE-ABS-KEY ("hybrid data") OR TITLE-ABS-KEY ("combined data") OR TITLE-ABS-KEY ("multimodal feature*") OR TITLE-ABS-KEY ("combined data")) AND (TITLE-ABS-KEY ("medical imag*") OR TITLE-ABS-KEY ("electronic health records") OR TITLE-ABS-KEY ("clinical data") OR TITLE-ABS-KEY ("MRI") OR TITLE-ABS-KEY ("fMRI") OR TITLE-ABS-KEY ("MR image*") OR TITLE-ABS-KEY ("sMRI") OR TITLE-ABS-KEY ("magnetic resonance imaging") OR TITLE-ABS-KEY ("EHR") OR TITLE-ABS-KEY ("CT scan*") OR TITLE-ABS-KEY ("X-ray") OR TITLE-ABS-KEY ("ultrasound") OR TITLE-ABS-KEY ("Mammogram") ) AND ( LIMIT-TO ( DOCTYPE,"ar" ) OR LIMIT-TO ( DOCTYPE,"cp" ) OR LIMIT-TO ( DOCTYPE,"ch" ) ) AND ( LIMIT-TO ( PUBYEAR,2022) OR LIMIT-TO ( PUBYEAR,2021) OR LIMIT-TO ( PUBYEAR,2020) OR LIMIT-TO ( PUBYEAR,2019) OR LIMIT-TO ( PUBYEAR,2018) OR LIMIT-TO ( PUBYEAR,2017) OR LIMIT-TO ( PUBYEAR,2016) OR LIMIT-TO ( PUBYEAR,2015) ) AND ( LIMIT-TO ( LANGUAGE,"English" ) )  -662 results | | |
| **Google Scholar** | | (“machine learning” OR “deep learning” OR “artificial intelligence” OR “reinforcement learning”) AND (“multimodal data” OR “fusion” OR "multimodality fusion" AND ("medical imag*" OR “electronic health records” OR “clinical”)  - Top 110 paper | | |
| **Embase** | |  | | |
| **#** | **Searches** | | **Results** |  |
| 1 | exp machine learning/ | | 283754 |  |
| 2 | "machine learning".mp. [mp=title, abstract, heading word, drug trade name, original title, device manufacturer, drug manufacturer, device trade name, keyword heading word, floating subheading word, candidate term word] | | 80430 |  |
| 3 | exp deep learning/ | | 20636 |  |
| 4 | "deep learning".mp. [mp=title, abstract, heading word, drug trade name, original title, device manufacturer, drug manufacturer, device trade name, keyword heading word, floating subheading word, candidate term word] | | 30653 |  |
| 5 | exp artificial intelligence/ | | 54442 |  |
| 6 | "artificial intelligence".mp. [mp=title, abstract, heading word, drug trade name, original title, device manufacturer, drug manufacturer, device trade name, keyword heading word, floating subheading word, candidate term word] | | 40755 |  |
| 7 | "neural network*".mp. [mp=title, abstract, heading word, drug trade name, original title, device manufacturer, drug manufacturer, device trade name, keyword heading word, floating subheading word, candidate term word] | | 95747 |  |
| 8 | "Convolutional neural network*".mp. [mp=title, abstract, heading word, drug trade name, original title, device manufacturer, drug manufacturer, device trade name, keyword heading word, floating subheading word, candidate term word] | | 19059 |  |
| 9 | "transfer learning".mp. [mp=title, abstract, heading word, drug trade name, original title, device manufacturer, drug manufacturer, device trade name, keyword heading word, floating subheading word, candidate term word] | | 2874 |  |
| 10 | "reinforcement learning".mp. [mp=title, abstract, heading word, drug trade name, original title, device manufacturer, drug manufacturer, device trade name, keyword heading word, floating subheading word, candidate term word] | | 5008 |  |
| 11 | "supervised learning".mp. [mp=title, abstract, heading word, drug trade name, original title, device manufacturer, drug manufacturer, device trade name, keyword heading word, floating subheading word, candidate term word] | | 4276 |  |
| 12 | "unsupervised learning".mp. [mp=title, abstract, heading word, drug trade name, original title, device manufacturer, drug manufacturer, device trade name, keyword heading word, floating subheading word, candidate term word] | | 2167 |  |
| 13 | "ensemble learning".mp. [mp=title, abstract, heading word, drug trade name, original title, device manufacturer, drug manufacturer, device trade name, keyword heading word, floating subheading word, candidate term word] | | 1408 |  |
| 14 | "Support vector machine".mp. [mp=title, abstract, heading word, drug trade name, original title, device manufacturer, drug manufacturer, device trade name, keyword heading word, floating subheading word, candidate term word] | | 32094 |  |
| 15 | "Artificial neural network*".mp. [mp=title, abstract, heading word, drug trade name, original title, device manufacturer, drug manufacturer, device trade name, keyword heading word, floating subheading word, candidate term word] | | 43975 |  |
| 16 | "decision tree".mp. [mp=title, abstract, heading word, drug trade name, original title, device manufacturer, drug manufacturer, device trade name, keyword heading word, floating subheading word, candidate term word] | | 19926 |  |
| 17 | "Random Forest".mp. [mp=title, abstract, heading word, drug trade name, original title, device manufacturer, drug manufacturer, device trade name, keyword heading word, floating subheading word, candidate term word] | | 18864 |  |
| 18 | "Naïve Bayes".mp. [mp=title, abstract, heading word, drug trade name, original title, device manufacturer, drug manufacturer, device trade name, keyword heading word, floating subheading word, candidate term word] | | 18 |  |
| 19 | "Naive Bayes".mp. [mp=title, abstract, heading word, drug trade name, original title, device manufacturer, drug manufacturer, device trade name, keyword heading word, floating subheading word, candidate term word] | | 2765 |  |
| 20 | "Fuzzy Logic".mp. [mp=title, abstract, heading word, drug trade name, original title, device manufacturer, drug manufacturer, device trade name, keyword heading word, floating subheading word, candidate term word] | | 5348 |  |
| 21 | "K- Nearest Neighbor*".mp. [mp=title, abstract, heading word, drug trade name, original title, device manufacturer, drug manufacturer, device trade name, keyword heading word, floating subheading word, candidate term word] | | 6343 |  |
| 22 | "K-means".mp. [mp=title, abstract, heading word, drug trade name, original title, device manufacturer, drug manufacturer, device trade name, keyword heading word, floating subheading word, candidate term word] | | 7412 |  |
| 23 | "Long Short-Term Memory Networks".mp. [mp=title, abstract, heading word, drug trade name, original title, device manufacturer, drug manufacturer, device trade name, keyword heading word, floating subheading word, candidate term word] | | 146 |  |
| 24 | "multimodal data".mp. [mp=title, abstract, heading word, drug trade name, original title, device manufacturer, drug manufacturer, device trade name, keyword heading word, floating subheading word, candidate term word] | | 660 |  |
| 25 | "late fusion".mp. [mp=title, abstract, heading word, drug trade name, original title, device manufacturer, drug manufacturer, device trade name, keyword heading word, floating subheading word, candidate term word] | | 114 |  |
| 26 | "joint fusion".mp. [mp=title, abstract, heading word, drug trade name, original title, device manufacturer, drug manufacturer, device trade name, keyword heading word, floating subheading word, candidate term word] | | 901 |  |
| 27 | "early fusion".mp. [mp=title, abstract, heading word, drug trade name, original title, device manufacturer, drug manufacturer, device trade name, keyword heading word, floating subheading word, candidate term word] | | 238 |  |
| 28 | "multimodality fusion".mp. [mp=title, abstract, heading word, drug trade name, original title, device manufacturer, drug manufacturer, device trade name, keyword heading word, floating subheading word, candidate term word] | | 81 |  |
| 29 | "multimodal imaging".mp. [mp=title, abstract, heading word, drug trade name, original title, device manufacturer, drug manufacturer, device trade name, keyword heading word, floating subheading word, candidate term word] | | 12482 |  |
| 30 | "hybrid data".mp. [mp=title, abstract, heading word, drug trade name, original title, device manufacturer, drug manufacturer, device trade name, keyword heading word, floating subheading word, candidate term word] | | 227 |  |
| 31 | "multimodal feature*".mp. [mp=title, abstract, heading word, drug trade name, original title, device manufacturer, drug manufacturer, device trade name, keyword heading word, floating subheading word, candidate term word] | | 150 |  |
| 32 | "combined data".mp. [mp=title, abstract, heading word, drug trade name, original title, device manufacturer, drug manufacturer, device trade name, keyword heading word, floating subheading word, candidate term word] | | 6052 |  |
| 33 | "medical imag*".mp. [mp=title, abstract, heading word, drug trade name, original title, device manufacturer, drug manufacturer, device trade name, keyword heading word, floating subheading word, candidate term word] | | 23475 |  |
| 34 | "electronic health records".mp. [mp=title, abstract, heading word, drug trade name, original title, device manufacturer, drug manufacturer, device trade name, keyword heading word, floating subheading word, candidate term word] | | 17538 |  |
| 35 | "clinical data".mp. [mp=title, abstract, heading word, drug trade name, original title, device manufacturer, drug manufacturer, device trade name, keyword heading word, floating subheading word, candidate term word] | | 159018 |  |
| 36 | MRI.mp. [mp=title, abstract, heading word, drug trade name, original title, device manufacturer, drug manufacturer, device trade name, keyword heading word, floating subheading word, candidate term word] | | 480382 |  |
| 37 | fMRI.mp. [mp=title, abstract, heading word, drug trade name, original title, device manufacturer, drug manufacturer, device trade name, keyword heading word, floating subheading word, candidate term word] | | 72812 |  |
| 38 | "MR image*".mp. [mp=title, abstract, heading word, drug trade name, original title, device manufacturer, drug manufacturer, device trade name, keyword heading word, floating subheading word, candidate term word] | | 28366 |  |
| 39 | sMRI.mp. [mp=title, abstract, heading word, drug trade name, original title, device manufacturer, drug manufacturer, device trade name, keyword heading word, floating subheading word, candidate term word] | | 753 |  |
| 40 | "magnetic resonance imaging".mp. [mp=title, abstract, heading word, drug trade name, original title, device manufacturer, drug manufacturer, device trade name, keyword heading word, floating subheading word, candidate term word] | | 976068 |  |
| 41 | EHR.mp. [mp=title, abstract, heading word, drug trade name, original title, device manufacturer, drug manufacturer, device trade name, keyword heading word, floating subheading word, candidate term word] | | 14104 |  |
| 42 | CT.mp. [mp=title, abstract, heading word, drug trade name, original title, device manufacturer, drug manufacturer, device trade name, keyword heading word, floating subheading word, candidate term word] | | 1215463 |  |
| 43 | X-ray.mp. [mp=title, abstract, heading word, drug trade name, original title, device manufacturer, drug manufacturer, device trade name, keyword heading word, floating subheading word, candidate term word] | | 596486 |  |
| 44 | ultrasound.mp. [mp=title, abstract, heading word, drug trade name, original title, device manufacturer, drug manufacturer, device trade name, keyword heading word, floating subheading word, candidate term word] | | 493078 |  |
| 45 | Mammogram.mp. [mp=title, abstract, heading word, drug trade name, original title, device manufacturer, drug manufacturer, device trade name, keyword heading word, floating subheading word, candidate term word] | | 7224 |  |
| 46 | 1 or 2 or 3 or 4 or 5 or 6 or 7 or 8 or 9 or 10 or 11 or 12 or 13 or 14 or 15 or 16 or 17 or 18 or 19 or 20 or 21 or 22 or 23 | | 369123 |  |
| 47 | 24 or 25 or 26 or 27 or 28 or 29 or 30 or 31 or 32 | | 20770 |  |
| 48 | 33 or 34 or 35 or 36 or 37 or 38 or 39 or 40 or 41 or 42 or 43 or 44 or 45 | | 6115229 |  |
| 49 | 46 and 47 and 48 | | 838 |  |
| 50 | limit 49 to (english language and yr="2015 -Current") | | 690 |  |
| 51 | limit 50 to exclude medline journals | | 156 |  |
