# Supplementary material for: Artificial intelligence-based methods for fusion of electronic health records and imaging data
Source: Sci Rep. 2022 Oct 26;12:17981. doi: 10.1038/s41598-022-22514-4 (PMC9605975; doi:10.1038/s41598-022-22514-4)
Supplement: Supplementary file 2 — Supplementary Information 2. [file 41598_2022_22514_MOESM2_ESM.docx]

**Appendix 2: Data Extraction Form**

| **Concept** | **Definition** |
| --- | --- |
| **Study Characteristics** |  |
| Author Name | The name of the first author of the study. |
| Year | The year in which the study was published. |
| Publication type | Show whether studies are Journal articles or Conference Proceedings |
| Country | The country of the first author’ institution. |
| Disease name | The disease that the study focuses on |
| Clinical outcome | The clinical task of the study. |
| Fusion strategy | The classification of the fusion approach used by each study (early, joint, late) |
| Feature extraction | The details related to feature extraction for imaging and EHR modalities in the studies. |
| **Dataset Characteristics** |  |
| Imaging type | The type of medical imaging used by studies |
| EHR type | The type of EHR used by studies |
| Data sources | Represent if data is freely available or not.  Public: All the data used in the study are publicly available.  Private: Data are not publicly available. |
| **AI characteristics** |  |
| AI branches | The branches/areas of AI used in the study, mostly categorized as machine learning, deep learning. |
| AI specific methods | The specific machine learning or deep learning models or algorithms used in the study. |
| **Evaluation** |  |
| Evaluation measures | The evaluation metrics used by the study to evaluate the performance of their fusion models. |
| Comparison with single  Modality models | Represent if the study compared the performance of its fusion model with single modality models |
